# Wearable Technologies for Gait Instability Rehabilitation: Mechanisms, Clinical Evidence, and Future Directions

**DOI:** 10.3390/bioengineering13091002

**Published:** 2026-08-28

**Authors:** Lijin Liu, Changfa Huang, Zhongyin Ji, Yujie Zhou, Zihua Li, Xueyi Zhang, Zhihong Wu

**Affiliations:** 1 Stem Cell Facility, Institute of Clinical Medicine, Peking Union Medical College Hospital, Chinese Academy of Medical Sciences and Peking Union Medical College, Beijing 100007, China; liulijin@aliyun.com (L.L.); lizihua8912@126.com (Z.L.); 2 State Key Laboratory of Complex Severe and Rare Diseases, Peking Union Medical College Hospital, Chinese Academy of Medical Sciences and Peking Union Medical College, Beijing 100007, China; 3 Department of Emergency Medicine, Beijing Jishuitan Hospital, Capital Medical University, Beijing 102208, China; cfh_7598@163.com; 4 Department of Orthopedic Surgery, Peking Union Medical College Hospital, Chinese Academy of Medical Sciences and Peking Union Medical College, Beijing 100007, China; zyji@zju.edu.cn (Z.J.); zhouyj2021@mail.sustech.edu.cn (Y.Z.); 5 National Infrastructures for Translational Medicine (PUMCH), Institute of Clinical Medicine, Peking Union Medical College Hospital, Chinese Academy of Medical Sciences and Peking Union Medical College, Beijing 100007, China

**Keywords:** gait instability, wearable rehabilitation, digital biomarkers, artificial intelligence, closed-loop systems

## Abstract

Wearable technologies are reshaping gait rehabilitation by shifting assessment and therapy from intermittent, clinic-based observation to continuous, data-driven, and adaptive care. This narrative review synthesizes the biomechanical basis of gait instability, the architecture and classification of wearable rehabilitation systems, and the clinical evidence for motion sensors, smart insoles, biofeedback devices, robotic and orthotic wearables, neuromodulatory systems, immersive platforms, and artificial intelligence (AI)-enabled closed-loop interventions. Its main contribution is an integrated framework that links AI, digital biomarkers, device components, adaptive control, and translational implementation, rather than treating wearable rehabilitation as a device-only or disease-specific topic. Current evidence indicates that these technologies can improve gait speed, symmetry, balance, endurance, fall-risk monitoring, and dual-task performance in neurological, musculoskeletal, frailty-related, and aging populations. However, the field is still limited by heterogeneous protocols, small samples, limited longitudinal validation, insufficient device standardization, usability barriers, cybersecurity concerns, uncertain reimbursement, and restricted interoperability with healthcare systems. Future progress will depend on multimodal sensor fusion, explainable and federated AI, digital twins, adaptive wearable robotics, tele-rehabilitation pathways, and large-scale pragmatic trials that validate effectiveness in real-world rehabilitation settings.

## 1. Introduction

From a biomechanical perspective, gait instability can be defined as a reduced capacity to maintain dynamic balance while the body’s center of mass moves over a continuously changing base of support [1,2,3]. It is reflected by increased stride-to-stride variability, abnormal step width, prolonged double-support time, reduced gait speed, impaired anticipatory and reactive postural adjustments, and reduced adaptability during turning, perturbation recovery, or dual-task walking [4,5,6,7,8]. Falls are therefore a clinically important consequence of unstable gait rather than a separate phenomenon, especially when impaired balance recovery is combined with reduced muscle power, delayed sensorimotor responses, environmental hazards, or cognitive load [9,10].

Gait instability occurs in older adults and in patients with neurological, musculoskeletal, and systemic conditions, including stroke, Parkinson’s disease (PD), spinal cord injury, and multiple sclerosis [11,12,13,14,15], as well as osteoarthritis, sarcopenia, frailty, peripheral neuropathy, and age-related sensory decline [15,16,17,18]. Its pathomechanical features vary by population: neurological disorders typically impair motor planning, rhythmicity, postural reflexes, and sensorimotor integration [2,3,11,12,13,14,15], whereas musculoskeletal disorders more often reduce joint range of motion, propulsion, alignment, pain-free loading, and limb symmetry [15,16,18]. During daily walking, these mechanisms often coexist and lead to compensatory strategies that increase energy expenditure and reduce walking safety.

Conventional gait rehabilitation remains the clinical foundation and includes therapist-guided balance training, strength conditioning, treadmill or overground task practice, cueing, neuromuscular re-education, and assistive devices [19,20,21,22,23,24]. Traditional orthoses and mobility aids, such as canes, walkers, ankle–foot orthoses, knee–ankle–foot orthoses, and shoe modifications, improve stability mainly through external support, alignment correction, joint constraint, or redistribution of load. Many modern wearable systems can be regarded as biomechanically adapted orthotic technologies: passive braces are being transformed into sensorized, powered, soft, or stimulation-driven devices that quantify movement, assist propulsion or foot clearance, and adjust support according to gait phase [25,26,27,28,29]. The central research gap is how to move from static support to adaptive assistance that is comfortable, clinically interpretable, and valid during daily-life walking.

Wearable technology refers to body-worn hardware and software systems that sense, process, transmit, interpret, and, in some cases, actively modify human movement. A typical gait-rehabilitation wearable contains inertial and motion-sensing modules, such as IMUs, accelerometers, gyroscopes, and magnetometers [30,31,32,33]. Plantar-pressure sensors and smart insoles provide foot-loading and center-of-pressure information [28,34,35], whereas surface electromyography can capture muscle activation patterns relevant to freezing of gait or abnormal motor control [36]. Some systems also incorporate EEG or fNIRS for neural monitoring during rehabilitation [11,37]. Wearable platforms further require local processing units or microcontrollers; wireless communication; power supply and battery management; embedded algorithms; user feedback interfaces; and optional actuators, such as motor drives, soft pneumatic or cable-driven assistance, functional electrical stimulation (FES), or vibrotactile cueing [26,27,29,38,39]. These components determine not only measurement accuracy but also comfort, adherence, safety, cost, and clinical scalability [33,40,41].

Wearable gait technologies can be classified by both structure and therapeutic function. Structurally, they include sensing, processing, communication, power, feedback, and actuation modules [33,35,41]. Functionally, they include passive monitoring and motion-sensing systems [33,35,42], feedback-driven cueing systems [38,43,44,45,46,47], robotic and exoskeletal wearables [27,29,39,48,49,50,51], FES-based systems [26,52,53], non-invasive neuromodulatory wearables [37,54,55,56,57,58], immersive and cognitive-integrated platforms [46,59,60,61,62,63], and AI-driven closed-loop rehabilitation systems [36,40,64,65]. Previous reviews have often focused on a single device class, one disease population, or sensor-based gait analysis [33,40,42]. In contrast, this review emphasizes the convergence of wearable hardware, digital biomarkers, AI analytics, adaptive control, clinical evidence, and translational barriers across rehabilitation contexts.

We propose that the clinical value of wearable gait rehabilitation depends on matching device architecture and control strategy to the patient’s pathomechanical deficit, rather than simply adding sensors to conventional therapy. The novelty of this review lies in linking gait pathomechanics, wearable-device architecture, AI-enabled digital biomarkers, closed-loop control, clinical evidence, and translational barriers within a single rehabilitation framework. Accordingly, this review aims to (i) summarize the biomechanical and pathophysiological basis of gait instability; (ii) classify wearable rehabilitation technologies by components, mechanisms, and clinical roles; (iii) synthesize evidence across neurological, musculoskeletal, frailty-related, and aging populations; (iv) discuss AI-enabled digital biomarkers and closed-loop rehabilitation; and (v) outline a roadmap for clinically scalable, personalized wearable rehabilitation ecosystems.

## 2. Materials and Methods

This article is a narrative review prepared with attention to the Scale for the Assessment of Narrative Review Articles (SANRA), particularly its emphasis on a clear rationale, transparent literature search, and structured synthesis. Because wearable gait rehabilitation spans engineering, rehabilitation medicine, neurology, orthopedics, gerontology, and digital health, a narrative design was selected to integrate heterogeneous device classes and clinical populations rather than to estimate a pooled treatment effect.

Literature searches were conducted in PubMed, Web of Science, Scopus, IEEE Xplore, and Google Scholar from database inception to July 2026. Search terms combined concepts related to gait instability and rehabilitation (“gait instability”, “gait disorder”, “fall risk”, “balance rehabilitation”, and “gait rehabilitation”); wearable technologies (“wearable sensor”, “inertial measurement unit”, “IMU”, “smart insole”, “pressure sensor”, “surface electromyography”, “exoskeleton”, “exosuit”, “orthosis”, “functional electrical stimulation”, “vibrotactile”, “virtual reality”, and “augmented reality”); intelligent systems (“artificial intelligence”, “machine learning”, “deep learning”, “digital biomarker”, “closed-loop”, “adaptive rehabilitation”, and “tele-rehabilitation”); and musculoskeletal or sports-related applications (“osteoarthritis”, “anterior cruciate ligament”, “sports injury”, “running gait”, “athlete”, and “injury prevention”). Reference lists of relevant reviews and clinical trials were also screened.

Eligible publications included clinical trials, pilot studies, validation studies, systematic or focused reviews, and representative engineering studies that reported wearable assessment or intervention for gait instability, balance impairment, fall risk, or gait rehabilitation. Studies were prioritized when they clearly described the target population, device components, intervention protocol, outcome measures, and clinically interpretable results. Evidence was categorized by device mechanism, including monitoring and sensing, feedback, robotics or orthoses, neuromodulation, immersive cognitive–motor platforms, and AI-enabled closed-loop systems. The synthesis focuses on clinical relevance, device architecture, translational barriers, and future research priorities. No formal meta-analysis was performed because of heterogeneity in device types, populations, interventions, and outcome measures.

## 3. Biomechanical and Pathomechanical Basis of Gait Instability

### 3.1. Pathomechanics of Gait Disorders in Neurological and Musculoskeletal Disease

Stable gait requires coordinated regulation of forward progression, limb clearance, weight acceptance, mediolateral balance, and adaptation to environmental constraints [2,3,66,67,68,69]. Pathomechanical gait instability emerges when one or more of these functions fails. Clinically relevant markers include slow gait speed, increased stride-time variability, reduced step length, prolonged double-support time, asymmetric stance or swing phases, impaired turning, reduced toe clearance, and delayed recovery after perturbation [67,70,71,72]. These gait features may reflect abnormalities in multisensory self-motion coding [68,73], cortical–basal ganglia–cerebellar control [74,75,76,77,78], spinal pattern generation [79], and cognitive–motor integration [71,72].

In neurological disease, instability is commonly driven by impaired motor planning, abnormal central rhythmicity, deficient postural reflexes, impaired proprioceptive integration, spasticity, weakness, or disrupted cognitive–motor control [2,3,67,72,75]. Stroke often produces asymmetric loading and reduced paretic propulsion [11,67]; PD is characterized by reduced automaticity, freezing episodes, short shuffling steps, and impaired turning [12,70,80]; cerebellar and vestibular disorders impair timing and sensory error correction [68,77,78,81]; and spinal cord injury disrupts descending control and spinal locomotor circuits [13,79,82].

In musculoskeletal and aging-related conditions, instability often arises from pain, joint deformity, reduced range of motion, sarcopenia, impaired lower-limb power, altered foot loading, and reduced capacity to absorb or generate joint moments. Knee or hip osteoarthritis, anterior cruciate ligament injury, peripheral neuropathy, and frailty may produce widened step width, reduced push-off, cautious gait, and increased energy cost [83,84,85,86]. These deficits explain why gait rehabilitation often requires both neuromotor retraining and biomechanical assistance.

Wearable systems are most clinically relevant when their sensing and actuation strategies are matched to specific pathomechanical deficits. For example, smart insoles can quantify plantar loading and stance asymmetry, IMUs can detect variability and turning instability, sEMG can capture abnormal muscle timing, FES can restore phase-specific dorsiflexion, and robotic orthoses can assist propulsion or limb clearance.

### 3.2. Mechanisms Underlying Wearable Rehabilitation

**Figure 1:**
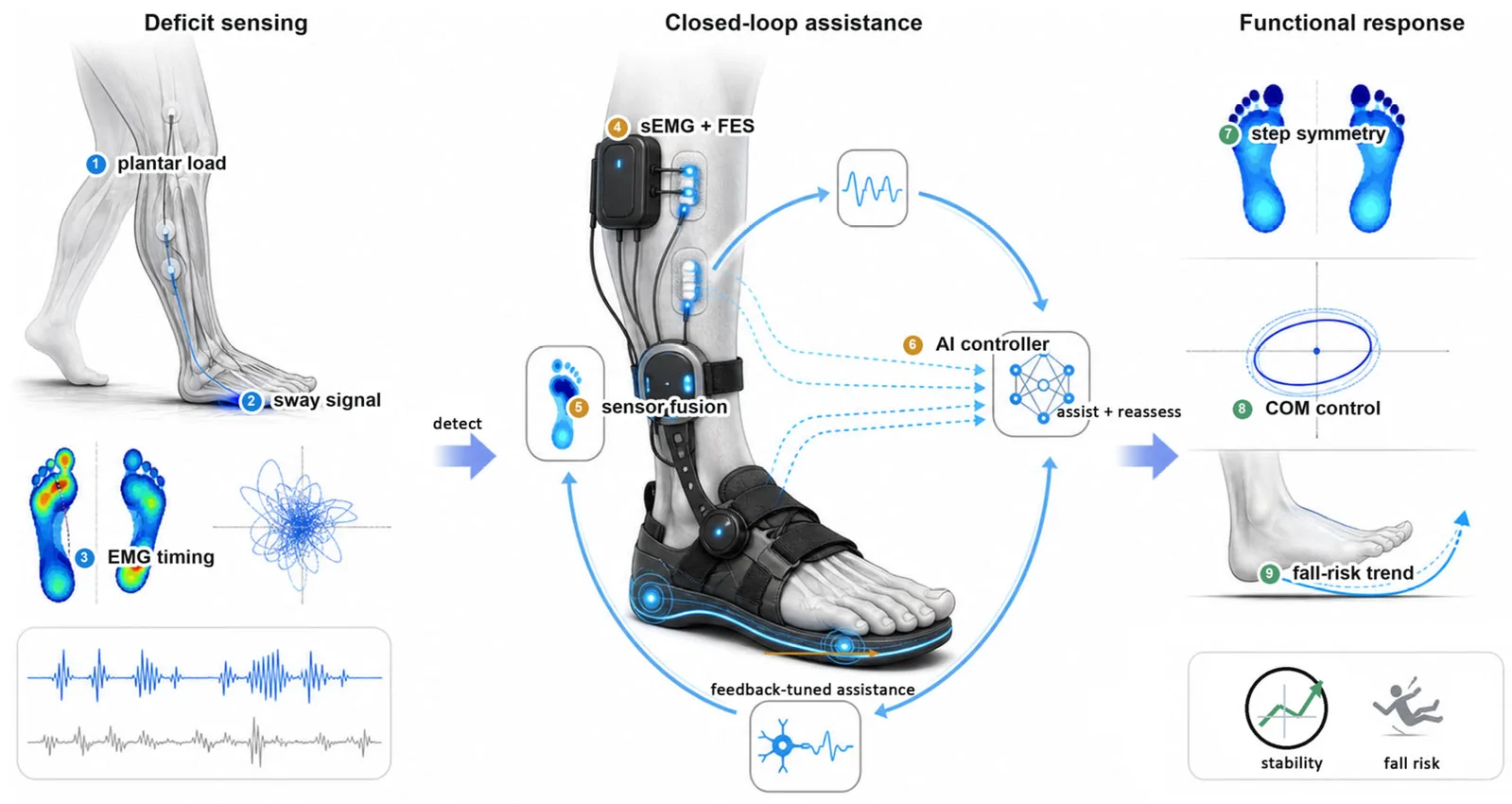
The key mechanisms of wearable-assisted rehabilitation.

The therapeutic rationale for wearable rehabilitation is based on its capacity to enhance sensorimotor integration, promote neuroplasticity, and support task-specific motor learning. Several complementary mechanisms underlie wearable-assisted rehabilitation (Figure 1).

First, wearable devices provide augmented sensory feedback that compensates for impaired internal motor cues. Visual, auditory, vibrotactile, or proprioceptive feedback can improve movement awareness and reinforce gait rhythmicity, particularly in disorders with impaired automatic motor control, such as Parkinson’s disease [38,43,44,45].

Second, wearable systems support intensive, repetitive, and task-specific training, which is essential for motor relearning and cortical reorganization [87]. Robotic exoskeletons and assistive devices can deliver high-dose locomotor practice while reducing the physical burden on therapists and patients [28,39,88].

Third, adaptive closed-loop systems continuously analyze movement performance and adjust stimulation, cueing, or mechanical assistance in real time [26]. This interaction supports error-based motor learning and individualized rehabilitation [26,34,64].

Fourth, wearable technologies extend gait assessment and intervention into real-world environments. Unlike laboratory-based assessments, wearable monitoring captures natural walking behavior during daily activities, thereby improving ecological validity and enabling long-term mobility tracking [28,46,59].

Together, these mechanisms position wearable technologies as key tools for precision gait rehabilitation and long-term mobility management.

## 4. Components and Classification of Wearable Technologies for Gait Rehabilitation

Wearable gait rehabilitation systems can be organized at two complementary levels: engineering architecture and therapeutic mechanism. At the architectural level, devices combine sensing, computation, communication, power management, feedback, actuation, and clinical interfaces. At the therapeutic level, they can be grouped into passive monitoring and motion-sensing systems, feedback-driven rehabilitation systems, robotic and exoskeletal wearables, electrical and neuromodulatory wearables, immersive and cognitive-integrated systems, and AI-driven closed-loop systems.

### 4.1. Wearable System Architecture and Device Components

A wearable rehabilitation device is not merely a single sensor. Most systems contain a sensing layer, an embedded processing layer, a communication layer, a power-management layer, and a human–device interface. Active systems may also include actuators, motor drivers, textile or rigid frames, electrode arrays, stimulation circuits, or haptic modules. Clinical deployment further requires software dashboards, data storage, cybersecurity controls, and interoperability with electronic health records or tele-rehabilitation platforms.

**Figure 2:**
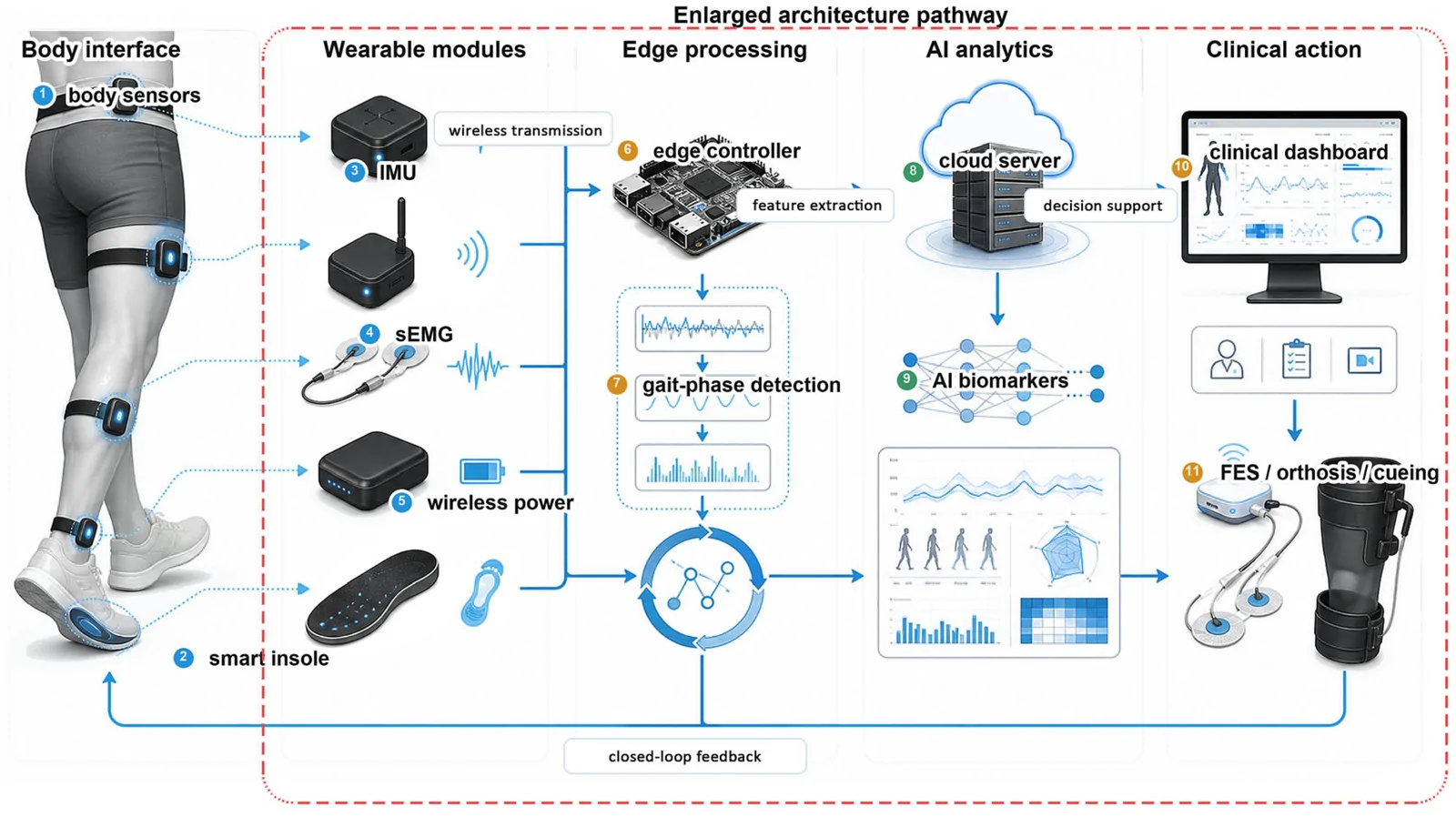
General architecture and component modules of wearable gait rehabilitation systems, including sensing, processing, AI analytics, control, actuation, power and communication support, and clinical interfaces.

Component selection should be driven by the target gait deficit. IMUs and accelerometers are suitable for gait-phase detection, cadence, turning, and stride variability; smart insoles and pressure sensors quantify plantar loading, center-of-pressure trajectories, stance symmetry, and foot progression; sEMG supports assessment of muscle timing and fatigue; EEG and fNIRS can monitor cortical engagement in cognitive–motor or neurofeedback paradigms; FES modules restore phase-specific muscle activation; and robotic actuators provide joint-level assistance or resistance. Figure 2 summarizes these component modules and their closed-loop relationships.

### 4.2. Passive Monitoring and Motion-Sensing Systems

Passive monitoring systems form the measurement foundation of wearable gait rehabilitation. IMUs, accelerometers, gyroscopes, magnetometers, smart insoles, plantar-pressure arrays, wearable force sensors, and sEMG systems can quantify gait speed, cadence, step length, symmetry, stride variability, turning, postural sway, muscle timing, and loading asymmetry [34,36,42,43,65]. These data streams support clinical assessment, home monitoring, and digital biomarker development beyond short laboratory walking tests.

Compared with observational scales, wearable sensors provide objective, high-frequency, and ecologically valid mobility data [33,89]. Smart insoles are particularly valuable because plantar-pressure distribution, center-of-pressure movement, and stance-phase loading reveal deficits that trunk-mounted sensors may miss [28,34]. Recent studies show that wearable sensors can estimate clinical mobility indices [89], detect freezing or near-loss-of-balance events [36,65], characterize gait patterns during exoskeleton-assisted rehabilitation [28], and support running or sports-related gait analysis [90].

These systems are increasingly being integrated into tele-rehabilitation frameworks. Their clinical value, however, depends on standardized sensor placement, calibration procedures, sampling frequencies, validation against reference systems, and interpretable reporting formats.

### 4.3. Feedback-Driven Rehabilitation Systems

Feedback-based wearable systems deliver real-time sensory cueing to guide movement correction and improve gait performance [47]. Common modalities include rhythmic auditory stimulation, visual cueing, vibrotactile feedback, and EMG-driven biofeedback. Auditory cueing provides rhythmic timing signals that enhance gait regularity and cadence synchronization, particularly in Parkinsonian gait disorders [43,45]. Visual cueing devices project spatial stepping targets that improve stride length and reduce freezing of gait [44], whereas vibrotactile systems mounted on the trunk or lower limbs provide haptic cues for postural correction and balance regulation [38]. Because many feedback systems are lightweight, relatively low-cost, and compatible with home use, they are attractive candidates for scalable rehabilitation.

### 4.4. Robotic, Orthotic, and Exoskeletal Wearables

Robotic, orthotic, and powered exoskeletal systems augment locomotion through rigid frames, soft textiles, cable drives, pneumatic actuators, electric motors, or hybrid orthosis–actuator designs. Traditional orthoses provide alignment and support, whereas powered ankle–foot orthoses, knee–ankle–foot orthoses, and overground robotic exoskeletons extend this role by adding sensors, gait-phase controllers, and adaptive assistance [27,39,48,49,50]. Soft exosuits provide a more compliant assistance strategy for propulsion and endurance training [29,51], and smart insoles can be integrated into exoskeleton-assisted rehabilitation for gait assessment [28]. These systems are particularly relevant for patients who require assistance with propulsion, foot clearance, weight-bearing practice, or high-dose repetitive stepping.

### 4.5. Electrical and Neuromodulatory Wearables

Neuromodulatory wearable systems apply electrical stimulation to peripheral nerves, spinal pathways, or cortical regions to enhance locomotor function. FES devices synchronize electrical impulses with gait phases to restore ankle dorsiflexion and improve foot clearance [26,52,53]. Non-invasive brain stimulation techniques, including transcranial direct-current stimulation (tDCS) and transcranial alternating-current stimulation (tACS), modulate cortical excitability and may facilitate motor learning [37,54,55,56,57]. Transcutaneous spinal cord stimulation and auricular vagus nerve stimulation may further enhance sensorimotor integration and gait rhythmicity [58]. Although clinical outcomes remain heterogeneous, combining neuromodulation with robotic or task-oriented rehabilitation may strengthen neuroplastic adaptation and locomotor recovery.

### 4.6. Immersive and Cognitive-Integrated Systems

Immersive rehabilitation platforms combine wearable technologies with virtual reality (VR), augmented reality (AR), and cognitive–motor training paradigms [60,61]. These systems create ecologically relevant training environments that engage motor, sensory, and executive neural circuits. VR- and AR-based interventions provide interactive visual feedback, dual-task training, and gamified rehabilitation experiences that may improve engagement and adherence [46,50,59,61,62]. Some systems integrate EEG or functional near-infrared spectroscopy to monitor cortical activation and adapt task difficulty [37,63]. By combining cognitive and motor training, immersive systems are particularly relevant for older adults [60,91], individuals with Parkinson’s disease [61,63], and patients with mild cognitive impairment [62,91].

### 4.7. AI-Driven and Closed-Loop Rehabilitation Systems

AI-enabled rehabilitation uses machine learning and deep learning to translate multimodal wearable data into clinically actionable decisions. Supervised models, including support vector machines, random forests, gradient boosting, and logistic regression, have been used to classify gait patterns, detect gait-phase transitions, estimate Timed Up-and-Go performance, and predict fall risk [36,64,65,89]. Deep-learning architectures, including convolutional neural networks, recurrent neural networks, long short-term memory networks, gated recurrent units, temporal convolutional networks, and transformers, are increasingly used for time-series recognition of freezing of gait, activity transitions, abnormal loading, and disease-specific motor states [36,64,65,92]. Reinforcement learning and adaptive control may further optimize robotic assistance [28,40], FES timing [26], cueing parameters, or therapy difficulty according to patient response [34,40].

Digital biomarkers derived from IMUs, smart insoles, sEMG, video, and physiological sensors can support personalized rehabilitation by tracking stride variability, asymmetry, turning quality, muscle activation, fatigue, balance confidence, and adherence during daily life. In a closed-loop system, sensing feeds a state-estimation model; the controller selects a therapeutic action; and the wearable delivers cueing, stimulation, or mechanical assistance. Subsequent performance data then update the model and therapy parameters. Current limitations include small and non-diverse datasets, limited external validation, inconsistent ground-truth labeling, weak interpretability, model drift across devices or disease stages, battery and edge-computing constraints, cybersecurity risks, and the possibility that biased training data could worsen inequities in older or underrepresented populations.

## 5. Results: Clinical Evidence Across Disease Populations

Clinical evidence was organized by target population, device or intervention, study design and sample, outcome measures, key findings, and interpretation (Table 1). The reviewed evidence includes neurological disorders, musculoskeletal and mixed rehabilitation cohorts, sports-related gait assessment, aging, frailty, and fall-prevention contexts; therefore, the scope is not limited to neurological disease.

### 5.1. Stroke Rehabilitation

Stroke is one of the most extensively studied conditions in wearable gait rehabilitation. Sensor-based cueing systems have been associated with improvements in gait symmetry, walking speed, balance, and endurance [33,45]. Robotic exoskeletons, robot-assisted gait training, and soft robotic suits support repetitive task-specific stepping and may improve lower-limb motor recovery or propulsion [29,49,50,51,94]. FES-based wearable systems are commonly used to improve foot clearance and gait-phase-specific muscle activation [26,52,53].

Several randomized controlled trials have reported that visual and auditory cueing systems improve spatiotemporal gait parameters and Berg Balance Scale scores when combined with treadmill training [45,95]. Robotic exoskeletons enable high-intensity repetitive gait practice and may improve lower-limb motor recovery, although superiority over dose-matched conventional therapy remains inconsistent [49,50,94]. Soft robotic exosuits have shown promising effects on gait endurance and propulsion symmetry during high-intensity training [29,51]. FES-based and peripheral stimulation protocols may improve foot clearance and gait recovery [26,52,53], whereas stimulation combined with augmented-reality treadmill training may further support gait automaticity and cortical adaptation [37].

Overall, wearable technologies appear most effective when they are embedded in intensive, task-specific, and multimodal rehabilitation programs.

### 5.2. Parkinson’s Disease

Parkinson’s disease is another major target population for wearable rehabilitation, particularly for freezing of gait, postural instability, and dual-task deficits [36,38,42,43]. Wearable visual, auditory, and augmented-reality cueing approaches are especially relevant for freezing and gait rhythmicity [44,46,61,63]. Neuromodulatory approaches are being explored for gait automaticity and cognitive–motor interference, including DLPFC tDCS [54], transcranial alternating-current stimulation [55], auricular vagus nerve stimulation [56], and cerebellar tDCS combined with augmented-reality treadmill training [37].

Visual and auditory cueing systems consistently improve stride length, cadence, and gait rhythmicity [44,47]. Augmented-reality dual-task platforms and wearable cueing devices have demonstrated non-inferior or superior performance compared with therapist-guided training in several randomized trials [44,46,61]. Wearable sensors also provide useful digital biomarkers for monitoring gait fluctuations, freezing episodes, and fall risk during daily life [36,64,65]. Neuromodulatory approaches may further improve gait automaticity and dual-task performance, including DLPFC tDCS [54], transcranial alternating-current stimulation [55], auricular vagus nerve stimulation [56], and cerebellar tDCS combined with augmented-reality treadmill training [37].

Integrating wearable cueing, cognitive training, and real-world mobility monitoring is likely to become increasingly important in Parkinsonian rehabilitation.

### 5.3. Cerebral Palsy

Wearable robotic systems have shown promising results in pediatric gait rehabilitation for cerebral palsy [39,59,96]. Overground robotic exoskeletons and wearable ankle-assist devices can improve gross motor function, gait symmetry, balance, and functional mobility [27]. Compared with conventional physiotherapy alone, robotic-assisted interventions may increase training intensity and engagement among children [27,96]. However, device size, weight, fitting complexity, growth-related adjustment, and long-term usability remain important barriers to pediatric deployment [39].

### 5.4. Spinal Cord Injury

Individuals with incomplete spinal cord injury may particularly benefit from robotic gait training combined with neuromodulation [48,82]. Exoskeleton-assisted locomotor training can improve walking endurance, balance, and functional mobility while enabling repetitive task-specific practice [48,88]. Recent evidence suggests that combining robotic rehabilitation with transcutaneous spinal stimulation or cortical stimulation may further enhance corticospinal excitability and locomotor recovery [88]. Nevertheless, current evidence remains limited by small sample sizes, short follow-up durations, and heterogeneous device configurations [28,48,88].

### 5.5. Aging, Frailty, and Fall Prevention

Wearable technologies are increasingly used in older adults for fall-risk assessment, balance training, and home-based rehabilitation [33,60,89,93]. Sensor-based monitoring can detect gait variability, turning instability, and postural sway associated with increased fall risk [33,89]. Immersive VR/AR systems and cognitive–motor dual-task training have improved balance, walking adaptability, and functional mobility in community-dwelling older adults [55,60,62,81,91]. Protective wearables, including smart airbags and orthotic systems, may also reduce injury severity during falls [93]. These technologies may ultimately support proactive mobility management and continuous fall prevention in aging populations.

### 5.6. Musculoskeletal and Sports-Related Rehabilitation

Although interventional trials remain more numerous in neurological rehabilitation, wearable technologies are also highly relevant to musculoskeletal and sports-related gait disorders. In osteoarthritis, anterior cruciate ligament injury, chronic pain, sarcopenia, and running-related injuries, IMUs, pressure sensors, smart insoles, and wearable EMG can quantify loading asymmetry, joint-protection strategies, altered foot progression, propulsion deficits, fatigue, and return-to-sport readiness [16,21,32,34,83,90]. This literature strengthens the rationale for including musculoskeletal populations in wearable gait rehabilitation, while also highlighting a gap: many sports and orthopedic studies emphasize assessment or injury-risk profiling, whereas fewer studies test adaptive wearable interventions with longitudinal clinical outcomes. Future trials should therefore evaluate whether sensor-derived biomechanical phenotypes can guide personalized orthotic tuning, feedback, exercise progression, and return-to-activity decisions.

## 6. Discussion: Clinical Translation, Barriers, and Generalizability

The evidence reviewed above supports the potential of wearable gait rehabilitation, but clinical effectiveness cannot be generalized simply by pooling device categories or disease labels. Studies differ in participant severity, chronicity, device configuration, therapist involvement, dosage, setting, outcome measures, and follow-up duration. Translation therefore requires careful consideration of methodological heterogeneity and real-world implementation barriers.

### 6.1. Generalizing Clinical Effectiveness Under Methodological Heterogeneity

More reliable generalization will require pragmatic multicenter trials; transparent reporting of rehabilitation dose; standardized core outcomes; and harmonized definitions of gait events, such as freezing, near-falls, falls, and successful community ambulation. Wearable-derived outcomes should be validated against laboratory reference systems and linked to patient-centered endpoints, including participation, confidence, caregiver burden, and fall-related injury. Stratified analyses are also needed because the same device may have different effects in early versus chronic stroke, tremor-dominant versus postural-instability PD, pediatric versus adult orthotic users, and frail older adults with multimorbidity.

### 6.2. Barriers to Widespread Clinical Adoption

Major adoption barriers include insufficient device standardization, inconsistent calibration, and heterogeneous signal-processing or outcome-reporting pipelines [33,35,40,41]. Limited interoperability with electronic health records and rehabilitation information systems further constrains clinical integration, particularly when wearable outputs are not translated into concise, interpretable information for clinicians [41,97,98]. Uncertain regulatory classification, inadequate reimbursement models, and limited clinician time to interpret continuous data streams also restrict adoption [40,99]. Wearable systems should therefore be developed with shared technical benchmarks and minimum validation requirements [33,40,41]; interoperable data formats [41,97]; and early evidence for safety, measurement validity, clinical benefit, cost-effectiveness, accessibility, and real-world usability [98,99,100].

Usability, cybersecurity, and long-term adherence are equally important for clinical translation [40,41,98]. In older adults and patients with neurological or musculoskeletal disability, device weight, donning and doffing difficulty, skin irritation, heat, cable management, charging burden, sensory impairment, cognitive load, fear of falling while wearing the device, and caregiver availability can all reduce adherence [40,60,98]. Because gait data may reveal sensitive information about mobility limitations, fall risk, disease progression, home routines, and activity patterns, secure data transmission, access control, consent, data ownership, and governance of secondary data use should be addressed before routine deployment [41,97]. For AI-enabled systems, external validation, bias assessment, model updating, uncertainty estimation, algorithmic transparency, and clinician override mechanisms are needed to maintain trust and prevent unsafe or inequitable recommendations [35,41,65].

### 6.3. Limitations of This Review

This review has several limitations. First, because it is a narrative review, it does not provide pooled effect estimates or formal risk-of-bias scoring for every included study. Second, although multiple databases and expanded search terms were used, relevant engineering prototypes, non-English studies, unpublished trials, or commercial device data may have been missed. Third, the literature remains heterogeneous with respect to populations, interventions, outcomes, and follow-up periods, limiting direct comparison. Finally, AI-enabled wearable rehabilitation is evolving rapidly; algorithms, regulatory expectations, and commercially available systems may change soon after publication.

## 7. Future Roadmap for Wearable Gait Rehabilitation

Future studies should move from isolated device demonstrations toward validated rehabilitation ecosystems. Priority components include modular multimodal sensing; age-friendly hardware; low-power edge computation; secure wireless communication; interoperable clinical dashboards; and adaptive controllers capable of delivering cueing, stimulation, or robotic assistance according to real-time gait state.

A practical roadmap includes seven priorities. First, multimodal sensor fusion should combine IMU, smart-insole, EMG, physiological, and contextual data to generate robust digital biomarkers. Second, digital twins of patient-specific gait mechanics may support individualized simulation and therapy prescription. Third, explainable AI should make predictions and controller decisions understandable to clinicians and patients. Fourth, federated learning and privacy-preserving analytics should enable multicenter model training without centralizing sensitive gait data. Fifth, tele-rehabilitation should connect home-based monitoring with clinician oversight and timely adjustment. Sixth, wearable robotics and neuromodulation should be integrated through adaptive closed-loop controllers. Seventh, large-scale longitudinal clinical trials and post-market studies should evaluate durability, adherence, safety, cost-effectiveness, and real-world fall reduction.

Future technology development should also define recommended component sets for different clinical use cases. Monitoring-focused systems should prioritize validated sensors, battery life, calibration stability, and interpretable dashboards. Intervention-focused systems should add safe actuation, adjustable assistance, fail-safe controls, and comfort-centered mechanical design. AI-enabled systems should include external validation, uncertainty estimation, cybersecurity safeguards, and mechanisms for clinician override.

## 8. Conclusions

Wearable gait rehabilitation is progressing from passive measurement toward AI-enabled, closed-loop, and clinically connected care. This review highlights that successful systems must align wearable components with specific biomechanical deficits, generate validated digital biomarkers, support adaptive intervention, and remain usable in real-world populations. Although current evidence supports improvements in gait performance, balance, endurance, and fall-risk monitoring, stronger clinical translation will require standardized methods, interoperable data systems, transparent AI, clearly defined reimbursement pathways and coverage policies, cybersecurity protections, and longitudinal trials. A future generation of multimodal, explainable, privacy-preserving, and human-centered wearable systems may help deliver personalized gait rehabilitation across neurological, musculoskeletal, frailty-related, and aging populations.

## Figures and Tables

**Table 1:** Clinical evidence for wearable gait rehabilitation technologies, organized by population, intervention, outcomes, and key findings.

Population	Device/Intervention	Study Design and Sample	Outcome Measures	Key Results and Clinical Interpretation	Ref.
Stroke with chronic foot drop	Peroneal nerve FES during gait training	Multicenter prospective RCT; *n* = 119; FES-assisted gait training versus conventional gait training.	Walking speed, gait status, FIM, and ankle movement.	FES improved walking ability and ankle control, supporting phase-synchronized stimulation as an adjunct for selected hemiplegic gait patterns.	[53]
Post-stroke gait impairment	Soft robotic exosuit during high-intensity gait training	Pilot study using exosuit-augmented high-intensity gait practice.	Walking endurance, training intensity, and propulsion symmetry.	Soft assistance increased feasible training intensity and showed preliminary improvement in endurance; larger dose-matched trials are needed.	[29]
Chronic stroke	Visual feedback plus rhythmic auditory cueing	RCT; *n* = 32; treadmill training with combined visual feedback and auditory rhythm.	Spatiotemporal symmetry, balance, and Berg Balance Scale.	Combined cueing improved gait symmetry and balance, supporting its role in motor timing and feedback-oriented relearning.	[45]
Stroke with hemiparetic gait	sEMG-controlled closed-loop FES	Closed-loop system using patient-derived sEMG from both limbs to trigger bilateral coordination.	FES timing precision, bilateral coordination, and adaptability.	Patient-driven control improved individual adaptability and illustrates a pathway toward intelligent closed-loop stimulation.	[26]
Subacute stroke	taVNS plus tDCS, alone or combined	Four-arm RCT; *n* = 169; combined stimulation, single-modality stimulation, or sham stimulation.	Gait function and neuromodulation response.	Combined taVNS and tDCS produced synergistic gait gains, although optimal dose and long-term durability require confirmation.	[57]
Parkinson’s disease	Wearable visual cueing	Randomized non-inferiority trial; wearable cueing versus therapist-guided training.	Gait and motor function.	Wearable cueing was non-inferior to therapist-guided training, supporting home-compatible cue delivery for PD gait rehabilitation.	[44]
Parkinson’s disease	AR dual-task rehabilitation	RCT evaluating an AR dual-task intervention.	Postural stability, functional mobility, and turning under single- and dual-task conditions.	AR dual-task practice improved postural stability and turning, addressing cognitive–motor interference in PD.	[61]
Parkinson’s disease with freezing of gait	AR cueing strategies	ELIMINATE FoG randomized trial comparing cueing approaches.	Freezing frequency and duration.	AR cueing reduced freezing; discrete cueing appeared particularly promising for personalized cue selection.	[46]
Parkinson’s disease	Wearable sEMG plus ML prediction	Wearable muscle-signal acquisition with machine-learning model development.	Prediction of freezing-of-gait episodes.	sEMG-derived features predicted freezing episodes, highlighting digital biomarkers for anticipatory intervention.	[36]
Children with cerebral palsy	Overground wearable robotic gait training	Multicenter single-blind RCT; *n* = 90; GMFCS II-IV; wearable robot versus conventional physiotherapy.	GMFM-88, balance, and gait pattern.	Wearable RAGT improved gross motor function, balance, and gait, although fit, growth, and usability remain pediatric constraints.	[27]
Children with CP or motor disorders	VR environment plus wearable haptics	Single-blind randomized crossover trial; *n* = 8.	Feasibility, motor function, and engagement.	Immersive haptic rehabilitation was feasible and engaging; evidence remains preliminary because of the small sample.	[59]
Spinal cord injury	Robotic gait training plus tDCS	Double-blind neurophysiology study with fNIRS monitoring.	M1/SMA cortical activity and locomotor recovery signals.	Active tDCS combined with RAGT increased cortical activation, supporting multimodal neuromodulation-robotics protocols.	[88]
Mixed neurological disorders	Smart insoles integrated with exoskeleton rehabilitation	Twelve-week program; *n* = 10 with stroke, SCI, TBI, or MS; smart-insole monitoring.	Gait phase, plantar pressure, and clustering-derived gait patterns.	Smart insoles quantified exoskeleton-assisted gait and addressed a literature gap in plantar-pressure monitoring during rehabilitation.	[28]
Older adults	Accelerometer-gyroscope fall-detection airbag	Algorithm and device test; *n* = 16 simulated falls.	Pre-impact detection time and deployment.	The algorithm detected fall signals before impact and triggered protection, but simulated falls limit real-world generalization.	[93]
Sarcopenia or frailty	Smart insoles plus AI digital biomarkers	Smart-insole plantar-pressure data from 83 participants analyzed using AI.	Pressure-derived gait biomarkers; sarcopenia classification.	Digital biomarkers distinguished sarcopenia from controls and support inclusion of smart insoles in wearable rehabilitation evidence syntheses.	[34]
Older adults	C-Mill VR/AR treadmill training	RCT; *n* = 60 adults aged 60–70 years; VR/AR treadmill training versus control.	Balance, fall risk, and functional mobility.	VR/AR treadmill training improved balance and reduced fall risk, supporting cognitively enriched gait training.	[60]

## Data Availability

No new data were created or analyzed in this study. Data sharing is not applicable to this article.
